# Evaluating the causal effects of life-course adiposity on jaw anomalies

**DOI:** 10.1186/s40510-025-00565-3

**Published:** 2025-05-16

**Authors:** Xin Chen, Zheng Cheng, Qianyi Wang, Yubin Jiang, Qing Cheng, Qianglin Jiang

**Affiliations:** 1 Department of Oral and Maxillofacial Surgery, Jiangyin People’s Hospital Affiliated to Nantong University, Jiangyin, China; 2 Department of Cardiology, Jiangyin People’s Hospital Affiliated to Nantong University, Jiangyin, China; 3 Department of Orthodontics, Jiangyin People’s Hospital Affiliated to Nantong University, Jiangyin, China

**Keywords:** Jaw abnormalities, Mandible, Retrognathia, Obesity, Mendelian randomization

## Abstract

**Background:**

Observational studies indicate that obesity correlates with jaw development and remodeling; however, causality remains unclear. This study aimed to examine the potential causal relationship between life-course adiposity and jaw anomalies.

**Methods:**

Utilizing summary statistics from genome-wide association studies predominantly of European ancestry, we conducted univariable and multivariable Mendelian randomization (MR) to estimate overall and independent effects of six obesity traits (birth weight, childhood body size, childhood body mass index [BMI], adult BMI, adult body fat percentage, and adult waist circumference) on seven jaw anomalies, including bimaxillary hypoplasia, prognathism, retrognathism, and jaw asymmetry. Comprehensive sensitivity analyses verified robustness, assessed heterogeneity, and examined pleiotropy.

**Results:**

In univariate analyses, genetically predicted thinner childhood body size (inverse variance weighted [IVW] OR: 0.41, 95% CI: 0.27–0.62, *p* < 0.001), adult BMI (IVW OR: 0.65, 95% CI: 0.53–0.80, *p* < 0.001), and waist circumference (IVW OR: 0.60, 95% CI: 0.45–0.82, *p* = 0.001) were significantly associated with the risk of mandibular retrognathia following Bonferroni correction. Multivariable MR analysis revealed a direct causal effect of childhood body size on mandibular retrognathia, independent of birth weight, adult adiposity, growth hormones, and lifestyle factors. No evidence was found for causal associations between life-course adiposity and other jaw anomalies. Sensitivity analyses produced broadly consistent findings.

**Conclusions:**

This MR study provides new evidence on the direct causal effects of thin childhood body size on the risk of mandibular retrognathia, emphasizing the critical role of early childhood nutrition and weight management in craniofacial development.

**Supplementary Information:**

The online version contains supplementary material available at 10.1186/s40510-025-00565-3.

## Background

Obesity has emerged as a major global health challenge affecting all segments of the population, including children and adolescents [1]. Notably, obesity is manifesting at younger ages, with approximately 8% of children under five, 33% of school-aged children, and 25% of youth aged 10–19 classified as overweight or obese [2]. Health sequelae include increased risks of cardiovascular disease, diabetes, psychosocial issues, and joint disorders in later life [3, 4]. From a life-course perspective, genetic and environmental factors influencing children’s growth may have lasting effects on adult health.

Beyond overall growth, obesity significantly impacts craniofacial development [5]. Excess weight in children is associated with early puberty onset, accelerated dental eruption, and cervical vertebrae maturation [6, 7]. In vivo studies demonstrate that the condyle cartilage cells are rich in leptin receptors, and exogenous leptin injection can promote anterior mandibular growth in lean mice [8]. Genetic evidence suggested the CC allele of rs8044769 in the FTO gene as a risk factor for temporomandibular osteoarthritis [9]. Notably, recent research highlights an increased risk of jaw anomalies, such as mandibular prognathism, among obese children and adolescents [5–7, 10]. They also display reduced mandibular plane angles and soft-tissue profile convexity [7]. Conversely, individuals with pronounced jaw anomalies may experience chronic feeding difficulties and appearance-related anxiety [11], both of which impact weight management. It is essential to note that observational studies on obesity’s effects on cranial development rely on small, homogenous samples, which may introduce confounding variables and potential reverse causality [5, 10]. To date, no systematic examination of the association between obesity and jaw anomalies has been conducted, leaving the causal role of obesity in jaw development unresolved.

Mendelian randomization (MR) has emerged as a powerful method for inferring causal relationships by leveraging genetic variations associated with specific exposures [4]. Because genotypes are determined at conception and remain unaffected by disease progression, MR substantially mitigates reverse causality risk. Prior studies using MR have suggested that a higher body mass index (BMI) is linked to increased mandibular bone miner density and a reduced risk of temporomandibular disorders [4, 12, 13]. The recent release of genome-wide association study (GWAS) data related to jaw anomalies (e.g., jaw hypoplasia, prognathism, retrognathism, and asymmetry) has provided robust genetic instruments for MR analysis [14]. Given the limitations of using a single BMI measure as a proxy for weight and body composition, incorporating additional adiposity indices, such as body size and body fat percentage, may yield a more comprehensive understanding of early-life obesity’s causal effects on jaw development. It is also essential to assess the effects of life-course adiposity on jaw anomalies due to age-related changes [15]. While childhood and adolescent obesity may directly impact jaw development, adult adiposity likely plays a critical role in bone remodeling [16]. Disentangling the impact of obesity at specific life stages is challenging, especially due to genetic correlations between childhood and adult adiposity. This complexity supports the use of a multivariable MR (MVMR) approach, which may reveal whether childhood adiposity has an independent or persistent influence on outcomes or if effects are mediated by other obesity traits. Notably, prior MVMR studies have suggested that childhood BMI, rather than adult BMI, causally associates with humerus length, tibiofemoral angle, and temporomandibular disorders [4, 12].

In the present study, we leveraged genetic instruments to genetically predict birth weight, childhood BMI, childhood body size, adult BMI, body fat percentage, and waist circumference as surrogate measures of obesity across various life stages, aiming to comprehensively estimate direct and indirect effects on jaw anomalies, including bimaxillary hypoplasia, prognathism, retrognathism, and jaw asymmetry. Our findings elucidate the causal relationship between life-course obesity and specific jaw anomalies and underscore the need for early preventive strategies.

## Methods

### Study design

**Fig. 1:**
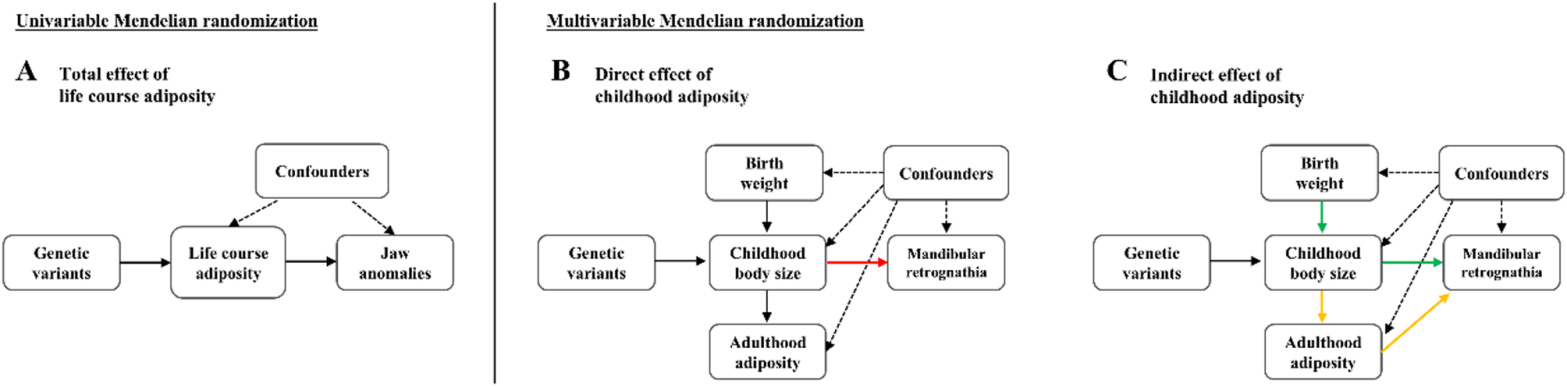
Overview of the present MR study. (A) The total effects of life-course adiposity (birth weight; childhood body mass index; childhood body size; adult body mass index; waist circumference; body fat percentage) on jaw anomalies (bimaxillary hypoplasia, prognathism, retrognathism, and jaw asymmetry) estimated by univariable MR; (B) The direct effect of childhood body size on mandibular retrognathia accounting for birth weight and adulthood adiposity estimated by multivariable MR; (C) Applying the same multivariable framework to estimate the indirect effects on mandibular retrognathia mediated along the causal pathway via birth weight or adulthood adiposity. The colored arrows in red, green, and orange on these graphs illustrate the causal effect of childhood body size on the outcome being estimated in multivariable MR analyses

The study design is illustrated in Fig. 1. Initially, we utilized human genetic data within the MR framework to explore the overall causal link between adiposity at various life stage and the risk of jaw anomalies (Fig. 1A). Subsequently, we quantified the specific effects of various obesity indicators on the likelihood of developing these anomalies (Fig. 1B, C). To reduce potential bias from population stratification, only individuals of European ancestry were included.

### Data source

#### Exposures

Five obesity traits were derived from the UK Biobank, a comprehensive biomedical database that includes health and genetic data for approximately 500,000 UK participants. The chosen traits included birth weight (*N* = 261,932), childhood body size (*N* = 453,169), adult BMI (*N* = 532,396), waist circumference (*N* = 407,661), and body fat percentage (*N* = 454,633). Birth weight (Kg) and childhood body size were self-reported by participants. Specifically, participants were asked to recall their body shape at age 10 compared to the average, categorizing themselves as “thinner”, “plumper”, or “above average” [17]. Those who selected “Do not know” or “Prefer not to answer” were excluded. Adult BMI was calculated by dividing body mass by the square of body height, and body composition was estimated via impedance measurement [18]. Additionally, childhood BMI GWAS data came from a meta-analysis involving up to 61,111 European children aged 2 to 10 years [19].

#### Outcomes

We sourced summary-level statistics on jaw anomalies from the FinnGen project (R11 version), where diagnoses were based on International Classification of Diseases codes K07.0 and K07.1 [14]. Jaw anomalies included both size-related anomalies, such as maxillary (104 cases, 453,629 controls) and mandibular hypoplasia (204 cases, 453,529 controls), and anomalies in jaw-cranial base relationship, including asymmetry of jaw (505 cases, 453,228 controls), maxillary prognathism (170 cases, 453,563 controls), mandibular prognathism (474 cases, 453,259 controls), maxillary retrognathism (598 cases, 453,135 controls), and mandibular retrognathia (2,354 cases, 451,379 controls). There was no sample overlap between obesity-related traits and outcomes in this MR study.

#### Covariates

To investigate the direct effects of specific life-course obesity on jaw anomalies, potential confounders were integrated into the MVMR models. These included growth hormone levels (*N* = 21,758), insulin-like growth factor 1 levels (*N* = 435,516), hypothyroidism (22,507 cases, 436,817 controls), moderate to vigorous physical activity levels (*N* = 377,234), and snoring (90,806 cases, 99,165 controls). Details of each research consortium, data type and source are provided in Table 1.

**Table 1:** Overview of the GWAS data used in the study

Phenotype	Consortium or cohort study	Sample size (cases/controls)	Ancestry	Data type	PubMed identifier	Data source
Exposure: life course adiposity						
Birth weight	UK Biobank	261,932	European	Continues	NA	https://gwas.mrcieu.ac.uk/datasets/ukb-b-13378/
Childhood body size	UK Biobank	453,169	European	Categorical Ordered	32376,654	https://gwas.mrcieu.ac.uk/datasets/ieu-b-5107/
Adult body mass index	UK Biobank	532,396	European	Continues	29892013	https://gwas.mrcieu.ac.uk/datasets/ebi-a-GCST90029007/
Waist circumference	UK Biobank	407,661	European	Continues	34017140	https://gwas.mrcieu.ac.uk/datasets/ebi-a-GCST90014020/
Body fat percentage	UK Biobank	454,633	European	Continues	NA	https://gwas.mrcieu.ac.uk/datasets/ukb-b-8909/
Childhood body mass index	EGG	61,111	European	Continues	33045005	https://www.ebi.ac.uk/gwas/publications/33045005
Confounding factors: hormones and lifestyles						
Growth hormone levels	Meta	21,758	European	Continues	33067605	https://gwas.mrcieu.ac.uk/datasets/ebi-a-GCST90012032/
Insulin-like growth factor 1 levels	UK Biobank	435,516	European	Continues	34226706	https://gwas.mrcieu.ac.uk/datasets/ebi-a-GCST90025989/
Hypothyroidism	UK Biobank	22,507/436,817	European	Binary	29892013	https://gwas.mrcieu.ac.uk/datasets/ebi-a-GCST90029022/
Moderate to vigorous physical activity levels	UK Biobank	377,234	European	Categorical Ordered	29899525	https://gwas.mrcieu.ac.uk/datasets/ebi-a-GCST006097/
Snoring	UK Biobank	90,806/99,165	European	Binary	32060260	https://gwas.mrcieu.ac.uk/datasets/ebi-a-GCST009762/
Outcome: jaw anomalies						
Major anomalies of jaw size						
Maxillary hypoplasia	FinnGen	104/453,629	European	Binary	36653562	https://storage.googleapis.com/finngen-public-data-r11/summary_stats/finngen_R11_K11_MAXIL_HYPOPL.gz
Mandibular hypoplasia	FinnGen	204/453,529	European	Binary	36653562	https://storage.googleapis.com/finngen-public-data-r11/summary_stats/finngen_R11_K11_MANDIBULAR_HYPOPL.gz
Anomalies of jaw-cranial base relationship						
Asymmetry of jaw	FinnGen	505/453,228	European	Binary	36653562	https://storage.googleapis.com/finngen-public-data-r11/summary_stats/finngen_R11_K11_ASYMMETRY_JAW.gz
Maxillary prognathism	FinnGen	170/453,563	European	Binary	36653562	https://storage.googleapis.com/finngen-public-data-r11/summary_stats/finngen_R11_K11_MAXIL_PROGN.gz
Maxillary retrognathism	FinnGen	598/453,135	European	Binary	36653562	https://storage.googleapis.com/finngen-public-data-r11/summary_stats/finngen_R11_K11_RETROG_MAXILLAE.gz
Mandibular prognathism	FinnGen	474/453,259	European	Binary	36653562	https://storage.googleapis.com/finngen-public-data-r11/summary_stats/finngen_R11_K11_MN_PROGN.gz
Mandibular retrognathia	FinnGen	2,354/451,379	European	Binary	36653562	https://storage.googleapis.com/finngen-public-data-r11/summary_stats/finngen_R11_K11_MN_RETRGN.gz

Abbreviation: EGG, Early Growth Genetics; NA, not available

#### Instrument selection

MR analysis requires that instrumental variables (IVs) fulfill three core assumptions: they must be strongly associated with the exposure, not related to confounders, and affect outcomes only through the exposure [20]. Briefly, single nucleotide polymorphisms (SNPs) were selected based on genome-wide significance (*p* < 5 × 10^−8^) to minimize false positives and underwent linkage disequilibrium clumping (r^2^ ≥ 0.001, clumping window ≤ 10,000 kb) to prove independent of each other. The *F*-statistic was calculated as we did before, with a value > 10 indicating reliable instrument strength [4, 21].

A total of 143 SNPs for birth weight, 17 for childhood BMI, 231 for childhood body size, 489 for adult BMI, 395 for body fat percentage, and 337 for waist circumference were identified, accounting for 1.5–6.0% of the variance in the life course adiposity (Supplementary Table 1). The minimum F statistic was 27, indicating that all IVs of the adiposity traits were sufficiently informative for MR analyses.

### Statistical analysis

#### Univariable MR (UVMR) and MVMR

The random-effects inverse variance weighted (IVW) method was applied in UVMR to estimate the total effect of obesity throughout life on jaw anomalies. Bonferroni correction addressed multiple testing, setting a significance threshold of 0.0012 (0.05/42). Associations with *p*-values between 0.01 and 0.001 were considered suggestive. Given the likely corrections among genetic determinants of obesity across life stages, MVMR models incorporated obesity traits at other life stages to determine independent effects. Additionally, the MVMR model was extended to adjust for hormone and lifestyle covariates, with the multivariable IVW (MV-IVW) served as the primary analysis method due to its high precision in estimating causal risks, assuming all IVs are valid.

#### MR sensitivity analysis

In UVMR, additional methods such as MR Egger, weighted median, and Mendelian randomized polymorphism RESidual Sum and Outlier (MR-PRESSO) were used to verify the robustness of IVW findings under varying assumptions. The weighted-median method can provide reliable causal effect estimates when fewer than half of the SNPs are invalid, whereas MR Egger offers robust inferences even if all instruments are compromised [22, 23]. Furthermore, MR Egger’s intercept term was analyzed to detect pleiotropic bias. MR-PRESSO could detect outlier SNPs indicative of horizontal pleiotropy and assess whether their exclusion impacts the causal estimates. For MVMR, MV-IVW results were validated with MVMR-median and multivariable MR-Egger (MVMR-Egger), and multivariable MR-Lasso (MVMR-Lasso) methods. The MVMR-Lasso approach employs lasso-type penalization on the direct effects of genetic variants on the outcome, generating post-lasso estimates via MV-IVW with only those genetic variants deemed valid by the lasso procedure [24]. Cochran’s Q test assessed IVs heterogeneity, and leave-one-out analysis checked for single SNP-driven effects.

IVW estimates were considered causal only if they demonstrated consistent direction and significance with at least one sensitivity analysis and showed no pleiotropy. All MR analyses were conducted using R (version 4.3.0) with the TwoSampleMR package (version 0.5.6), MRPRESSO (version 1.0), and MendelianRandomization (version 0.7.0).

## Results

### Total effects of life course adiposity on jaw anomalies

**Fig. 2:**
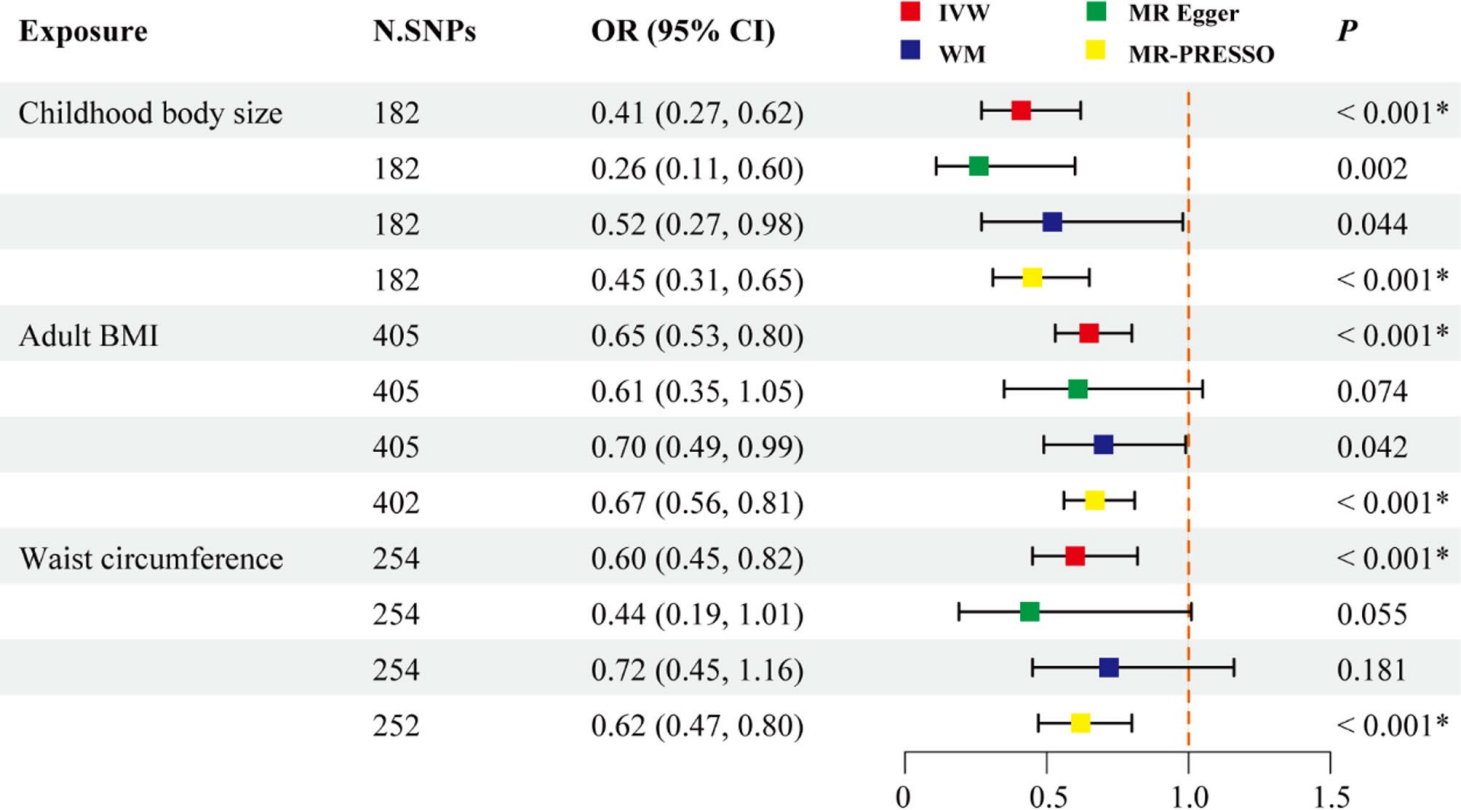
Forest plot depicting MR results for the association of genetically proxied life-course adiposity with mandibular retrognathia. Abbreviations: N. SNPs, number of SNPs used in MR; OR, odds ratio; CI, confidence intervals; IVW, inverse variance weighted; WM: weighted median; BMI: body mass index. *, *P* < 0.05/42

Applying a Bonferroni correction of 0.0012(0.05/42) to adjust for multiple tests, a total of 3 significant obesity-trait associations were observed (Fig. 2). Specifically, we found that a reduced risk of mandibular retrognathia associated with childhood body size (IVW OR: 0.41, 95% CI: 0.27–0.62, *p* < 0.001), adult BMI (IVW OR: 0.65, 95% CI: 0.53–0.80, *p* < 0.001), and waist circumference (IVW OR: 0.60, 95% CI: 0.45–0.82, *p* = 0.001). These associations were directionally consistent across sensitivity analyses and robust under the MR-PRESSO approach. For these significant associations, diagnostic tests (MR-Egger intercept ≤ 0.008, *p* ≥ 0.219) and visual detections via funnel plots, demonstrated no evidence of bias from pleiotropy (Supplementary Table 2, Supplementary Fig., 2). However, variant heterogeneity tests indicated potential heterogeneity across these associations (*p* < 0.001). Leave-one-out analysis confirmed that no single SNP significantly drove the causal associations between adiposity traits and mandibular retrognathia (Supplementary Fig. 3).

Genetically proxied childhood BMI (IVW OR: 0.68, 95% CI: 0.46–0.99, *p* = 0.045) and body fat percentage (IVW OR: 0.71, 95% CI: 0.53–0.95, *p* = 0.023) demonstrated a trend toward association with mandibular retrognathia but did not reach the corrected significance threshold (Supplementary Table 2). Birth weight was found to have a potential causal effect on mandibular prognathism (IVW OR: 2.34, 95% CI: 1.33–4.12, *p* = 0.003). No causal association was detected between life course adiposity and maxillary hypoplasia, mandibular hypoplasia, maxillary retrognathism and prognathism (all IVW *p* > 0.05).

### Direct effect of childhood body size on mandibular retrognathia

**Fig. 3:**
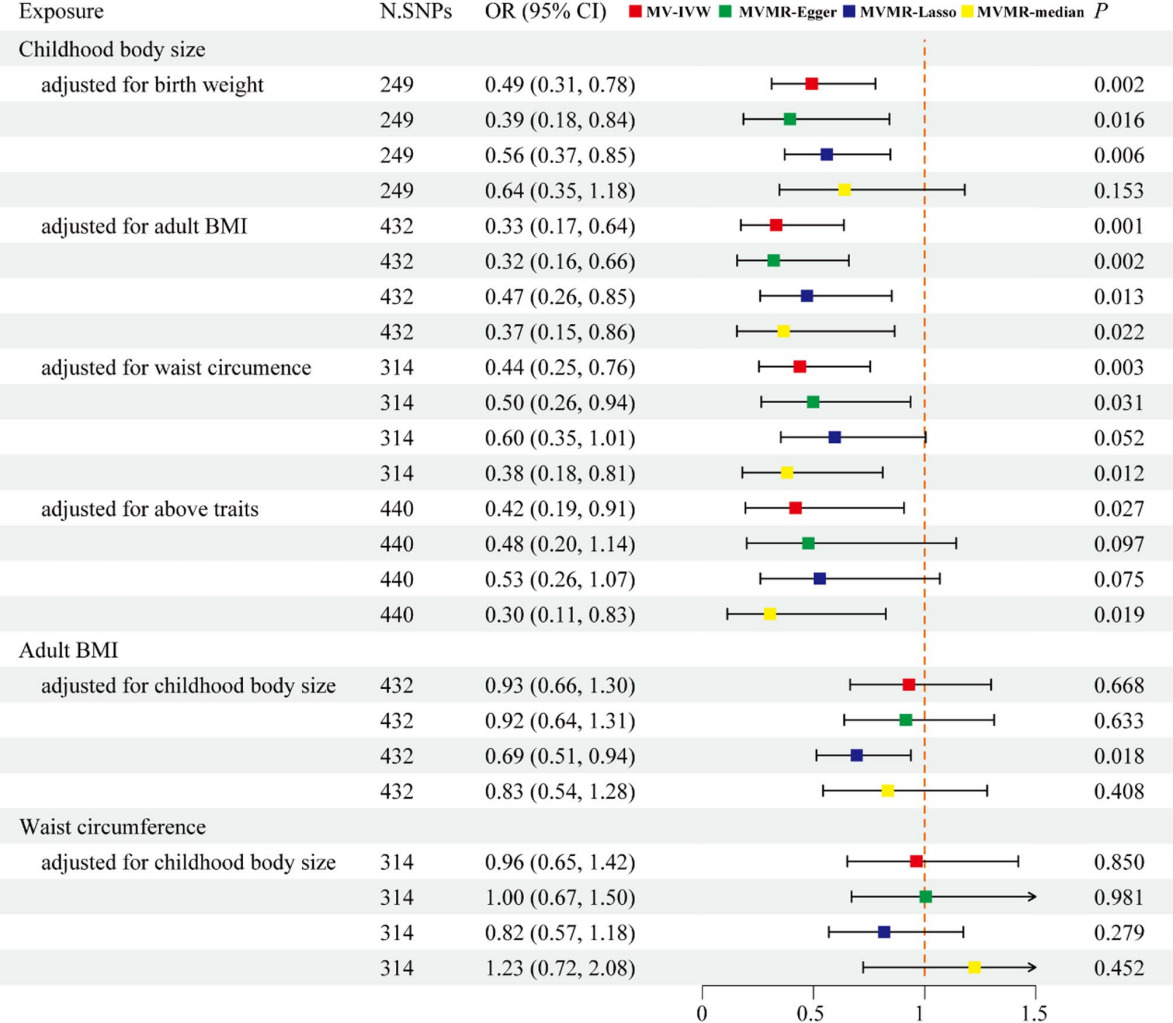
Multivariable MR estimating the association of birth weight, childhood body size and adult adiposity with mandibular retrognathia. Abbreviations: N. SNPs, number of SNPs used in MR; OR, odds ratio; CI, confidence intervals; MV-IVW, multivariable inverse-variance weighted; MVMR, multivariable Mendelian randomization; BMI, body mass index

In the MV-IVW analyses, the effect of childhood body size on the reduced risk of mandibular retrognathia remained significant and direct after adjusting for birth weight (OR: 0.49, OR: 0.31–0.78, *p* = 0.002), adult BMI (OR: 0.33, 95% CI: 0.17–0.64, *p* = 0.001), and waist circumference (OR: 0.44, 95% CI: 0.25–0.76, *p* = 0.003), or all factors combined (OR: 0.42, 95% CI: 0.19–0.91, *p* = 0.027) (Fig. 3, Supplementary Table 3). However, the associations between mandibular retrognathia and genetically predicted adult BMI (MV-IVW OR: 0.93, 95% CI: 0.67–1.30, *p* = 0.668) and waist circumference (MV-IVW OR: 0.96, 95% CI: 0.65–1.42, *p* = 0.850) disappeared after adjusting for childhood body size. Following additional adjustments for growth hormone levels, insulin-like growth factor 1 levels, presence of hypothyroidism, physical activity and snoring, all four multivariable methods continued to support the causal relationship between childhood body and mandibular retrognathia (Supplementary Table 4). Cochran’s Q test indicated heterogeneity across instrumental variables, while MVMR-Egger intercept tests revealed minimal pleiotropy (Supplementary Tables 3, 4).

## Discussion

To data, this is the first study to investigate the causal relationship between life-course adiposity and jaw anomalies using complementary MR approaches. Our analysis provides robust evidence that genetically predicted obesity traits at various life stages exert a total effect on anomalies related to mandibular retrognathia, but not on major jaw size anomalies. Additionally, when applying MVMR to account for adiposity on other life stages and various confounding factors (e.g., growth hormones and lifestyles), only the effects of childhood body size on mandibular retrognathia remained significant and direct. Besides, there was limited evidence supporting a direct or indirect role of other life-course adiposity factors, such as birth weight, adult BMI, body fat percentage and waist circumference, in causing jaw anomalies [25].

Our findings align with previous research highlighting the association between obesity and craniofacial development. It is well-established that pediatric individuals with obesity exhibit earlier growth onset, larger final skeletal dimensions, and an increased incidence of bimaxillary prognathism [5, 6, 10]. However, most previous studies have focused on imaging measurements, often encountering difficulties in identifying suitable control groups due to ethical constraints limiting radiographic exposure of non-patients. Longitudinal cephalometric growth data from normal populations serving as controls remain scare. A prospective study by Al-Taai et al. explored craniofacial changes, including skeletal, soft tissue, and dental modifications, in untreated orthodontic subjects with normal occlusion from ages 13 to 62 [15]. Interestingly, bimaxillary changes could persist into the sixth decade of life, characterized by substantial posterior rotation of the mandible and retrognathism from ages 31 to 62 [15]. Most observational studies have predominantly focused on children and adolescents, neglecting the potential impact of birth weight and adult obesity on jaw development and remodeling throughout the lifespan [5, 6]. Our study addresses critical gaps in the literature by specifically examining the role of life-course adiposity in jaw anomalies, thus contributing to a more comprehensive understanding of the long-term effects of obesity on jaw morphology. Not surprisingly, our findings did not demonstrate causal effects of life-course obesity on maxillary and mandibular hypoplasia, a result that appears rational given that facial proportions did not exhibit significant deviations from normality, while dentofacial dimensions were relatively greater in obese individuals [7, 10].

Hormonal and environmental factors play important roles in the development of mandibular retrognathia. Individuals with higher socioeconomic status often have better access to educational and healthcare resources, including specialized dental care, which may facilitate early diagnosis and timely intervention for craniofacial anomalies [26]. Additionally, certain oral habits, such as habitual chin resting, mouth breathing, and thumb sucking, have been implicated in mandibular retrognathia by altering muscle function and mechanical forces on the mandible [27]. From a hormonal perspective, growth hormone and insulin-like growth factor 1 are key regulators of mandibular development, primarily by stimulating chondrocyte proliferation in the condylar cartilage [28]. Deficiencies in growth hormone and insulin-like growth factor 1 have been associated with impaired mandibular growth, potentially contributing to retrognathism. Similarly, thyroid hormone imbalances can disrupt endochondral ossification in the condyle, further impacting mandibular projection [29]. However, the association between childhood body size and mandibular retrognathia remained significant even after adjusting for growth hormone levels, insulin-like growth factor 1 levels, presence of hypothyroidism, physical activity and snoring.

In this study, we employed objective measurements of childhood BMI and self-reported body size at age of 10 as indicators of childhood obesity. Our study may contribute to the field by suggesting a potential link between childhood obesity and a reduced risk of mandibular retrognathia in adulthood, a hypothesis indirectly supported by previous observational studies [5, 7]. This insight could have important implications for early orthodontic intervention and skeletal surgery. Although the precise mechanisms linking childhood body size and mandibular retrognathia remain unclear, several plausible pathways have been proposed. One potential mechanism is that underweight children may experience alternations in temporomandibular structure and function during a critical period of craniofacial development, which in turn inhibit the anterior and inferior growth of the mandible [4, 30]. The condyle plays a critical role in mandibular development, as its adaptive growth is influenced by both mechanical loading and functional stimuli. Previous studies have indicated that children with a higher BMI may be at a lower risk of temporomandibular disorders than those with a lower BMI [4]. This association may be attributed to improved masticatory efficiency and reduced chewing frequency, which may reduce excessive mechanical strain on the temporomandibular joint (TMJ) [31]. In contrast, individuals with a lower BMI may be more susceptible to temporomandibular dysfunction due to increased functional strain on the joint [31]. Magnetic resonance imaging studies have demonstrated that patients with TMJ pain frequently exhibit increased signal intensity in the bilaminar tissue, a finding indicative of hypervascularity and attachment edema [32, 33]. These structural alterations may contribute to progressive mandibular growth disturbances. If left untreated, temporomandibular disorders might result in significant facial growth impairment, including mandibular retrognathia or facial asymmetry [33]. Additionally, upper airway narrowing and tongue hypertrophy, often associated with obesity, may further promote forward displacement of the mandible to maintain airway patency [34]. Furthermore, leptin, a hormone that enhances muscle mass and strength, may be elevated in obese individuals, leading to increased tension in the masseter muscle and promoting a more upward and anterior mandibular position in both vertical and horizontal dimensions [35].

Given the critical periods for bone development (e.g., childhood and adolescence), the impact of obesity prior to early adulthood on craniofacial morphology is particularly relevant for orthodontic treatment. However, due to age-related changes in jaw morphology and the demographic characteristics of our outcome population (mean age 41.5) [14], GWAS data on adult obesity were also incorporated into our MR analysis. Our findings indicate an inverse relationship between adult obesity (e.g., BMI and waist circumference) and mandibular retrognathia, aligning with the results of two retrospective cohort studies conducted in Chinese and French adult populations [34, 36]. However, neither these observational studies nor our MR analysis accounted for age-related oral issues impacting craniofacial morphology, such as periodontitis, caries, and tooth loss. Additionally, the SNB angle, which reflects mandibular position in the sagittal dimension, gradually increases during puberty and subsequently declines in late adulthood [15]. Researchers have suggested that the observed mandibular retrognathia in adulthood may be attributed to age-related reductions in cortical bone thickness [37]. Furthermore, when adjusting for childhood body size in MVMR, the influence of adult adiposity on mandibular retrognathia disappeared. Our findings suggest that the observed negative associations between adult obesity and jaw anomalies may be influenced by a history of lower childhood BMI or by the inclusion of a significant proportion of young adults within the sample [34, 36]. On the other hand, birth weight, which is significantly affected by maternal nutrition and health, might not accurately reflect an individual’s susceptibility to diseases in adulthood [38].

Multidisciplinary strategies are essential for addressing jaw anomalies. This study provides novel evidence that children with lower body weight are at a higher risk of developing mandibular retrognathia. These findings underscore the importance of early nutritional support and growth education to prevent adverse oral habits and promote optimal craniofacial development. Addressing childhood undernutrition within public health initiatives could reduce the prevalence of jaw anomalies, with broad societal implications. Clinically, this study emphasizes the necessity of incorporating weight assessment into routine orthodontic evaluations for children and adolescents. Evidence indicates the childhood overweight accelerates dental and skeletal maturation, influencing orthodontic treatment planning, including the timing of serial extractions and growth modification [39]. However, recent surveys indicate that 55% of orthodontists fail to document weight-related data, and nearly 73% do not assess obesity during patient evaluations [40]. These findings advocate for a paradigm shift in clinical practice, making weight monitoring a standard component of orthodontic care. Integrating weight management strategies into orthodontic practice could enhance treatment outcomes and overall health for children and adolescents, especially for those with low weight and mandibular retrognathia. Our study leveraged summary GWAS data, where childhood body size was genetically proxied based on self-reported recall of body shape at age 10. Due to data limitations, stratification by specific age groups or weight categories was not feasible. We observed a significant inverse association between childhood body size and mandibular retrognathia, whereas childhood BMI (ages 2 to 10) showed only a suggestive trend without reaching statistical significance. These findings suggest that a leaner body size in late childhood may predispose individuals to mandibular retrognathia, reinforcing the need for early nutritional intervention. Public health initiatives should prioritize addressing childhood undernutrition, ensuring adequate caloric intake, high-quality protein, and essential nutrients—such as calcium, vitamin D, and omega-3 fatty acids—to support bone growth and craniofacial development [41]. In clinical practice, dentists, particularly orthodontists, should collaborate with nutritionists to incorporate weight management strategies into treatment plans, ensuring comprehensive care and mitigating growth-related complications linked to abnormal body weight.

While this study provides valuable insights, further research is necessary to validate these findings. Longitudinal studies are essential to track the effects of childhood body weight over time and its long-term impacts on craniofacial development. This would help establish causal relationships and better understand the mechanisms linking early-life adiposity to jaw anomalies. Additionally, the current findings need to be validated in more diverse populations, including different age groups, ethnicities, and geographic regions, to ensure that the results are generalizable and not influenced by cultural or demographic factors. Finally, understanding the biological mechanisms behind the relationship between early-life adiposity and jaw anomalies is crucial. Research into hormonal and genetic factors influencing both obesity and craniofacial development could help refine the underlying theories and inform more effective prevention strategies.

Several limitations should be considered when interpreting our findings. Firstly, the MR analysis was conducted exclusively on European samples, which may limit the applicability of the findings to other racial and ethnic groups. Secondly, it is important to note that all significant associations identified in our study were based on self-reported body size at age 10. Self-reported measures are inherently prone to misclassification bias, as they rely on individual recall and subjective interpretation, which could lead to overestimation or underestimation of adiposity. This misclassification may distort the true relationship between childhood obesity and craniofacial development, potentially leading to misleading conclusions regarding the strength and direction of these associations. However, the large sample size (over half a million) and the population-based design of the UK Biobank help mitigate some of the recall bias typically associated with self-reported data [42]. The substantial sample size reduces the impact of individual variability and enhances the robustness of the estimated relationship between childhood body size and jaw anomalies. Furthermore, it is important to knowledge that self-reported data do not account for fat distribution, which could be crucial in understanding how adiposity affects craniofacial structures in contrast to other body regions. Thirdly, it is well established that BMI is an imperfect measure of body fatness in pediatric populations, especially in children under the age of 5 years [43]. In this study, BMI data were collected from children aged 2 to 10 years, with a mean age of 7 years [19]. The use of BMI as a proxy for adiposity may have led to potential misclassification of children’s obesity status, biasing the observed relationships between childhood BMI and jaw anomalies. To address this limitation, future studies should consider incorporating alternative adiposity measures, such as the waist-to-height ratio or skinfold thickness, which could provide a more accurate understanding of the role of childhood obesity in shaping long-term health outcomes, including craniofacial anomalies. However, it is important to note that the available GWAS data on childhood obesity are currently limited to body size and BMI, which restricts the inclusion of other adiposity measures [13, 20]. Fourthly, although we adjusted for certain hormones and lifestyle factors in the MVMR analysis, other potential confounding factors, such as socioeconomic status education level and dietary habits, may have been overlooked. Insufficient control of these confounding variables could introduce bias or inaccuracies in MR estimates. Lastly, mandibular retrognathia is typically identified and managed with non-surgical interventions in children aged 4 to 10 [44]. Given that the FinnGen sample has a mean age of 41.5 years, some participants may have previously undergone orthodontic or surgical treatments for jaw deformities. Consequently, the causal effects of obesity on jaw anomalies may be underestimated in our study.

## Conclusions

This MR study provides new evidence on the direct causal effects of thin childhood body size on the risk of mandibular retrognathia, emphasizing the critical role of early childhood nutrition and weight management in jaw development. Dentists should pay more attention to underweight children when seeking medical advice for mandibular retrognathia. Future research should refine these findings by utilizing more accurate measures of adiposity and validating the results in diverse populations to improve generalizability. Longitudinal studies exploring the mechanisms linking early-life adiposity with jaw anomalies are also warranted.

## Electronic supplementary material

Supplementary Material 1.

Supplementary Material 2.

## Data Availability

All data generated or analyzed during this study are included in supplementary material or in the data repositories listed in the methods.

## Abbreviations

MR: Mendelian randomization
BMI: Body mass index
GWAS: Genome-wide association study
MVMR: Multivariable mendelian randomization
IVs: Instrumental variables
SNPs: Single nucleotide polymorphisms
UVMR: Univariable mendelian randomization
IVW: Inverse variance weighted
MV-IVW: Multivariable inverse variance weighted
MR-PRESSO: Mendelian randomized polymorphism RESidual Sum and Outlier
