# Supplementary material for: Evaluating the causal effects of life-course adiposity on jaw anomalies
Source: Prog Orthod. 2025 May 16;26:18. doi: 10.1186/s40510-025-00565-3 (PMC12084473; doi:10.1186/s40510-025-00565-3)
Supplement: Supplementary file 2 — Supplementary Material 2. [file 40510_2025_565_MOESM2_ESM.docx]

Supplementary Fig. 1 Scatter plot of the causal relationship between life-course adiposity with mandibular retrognathia using different MR methods.


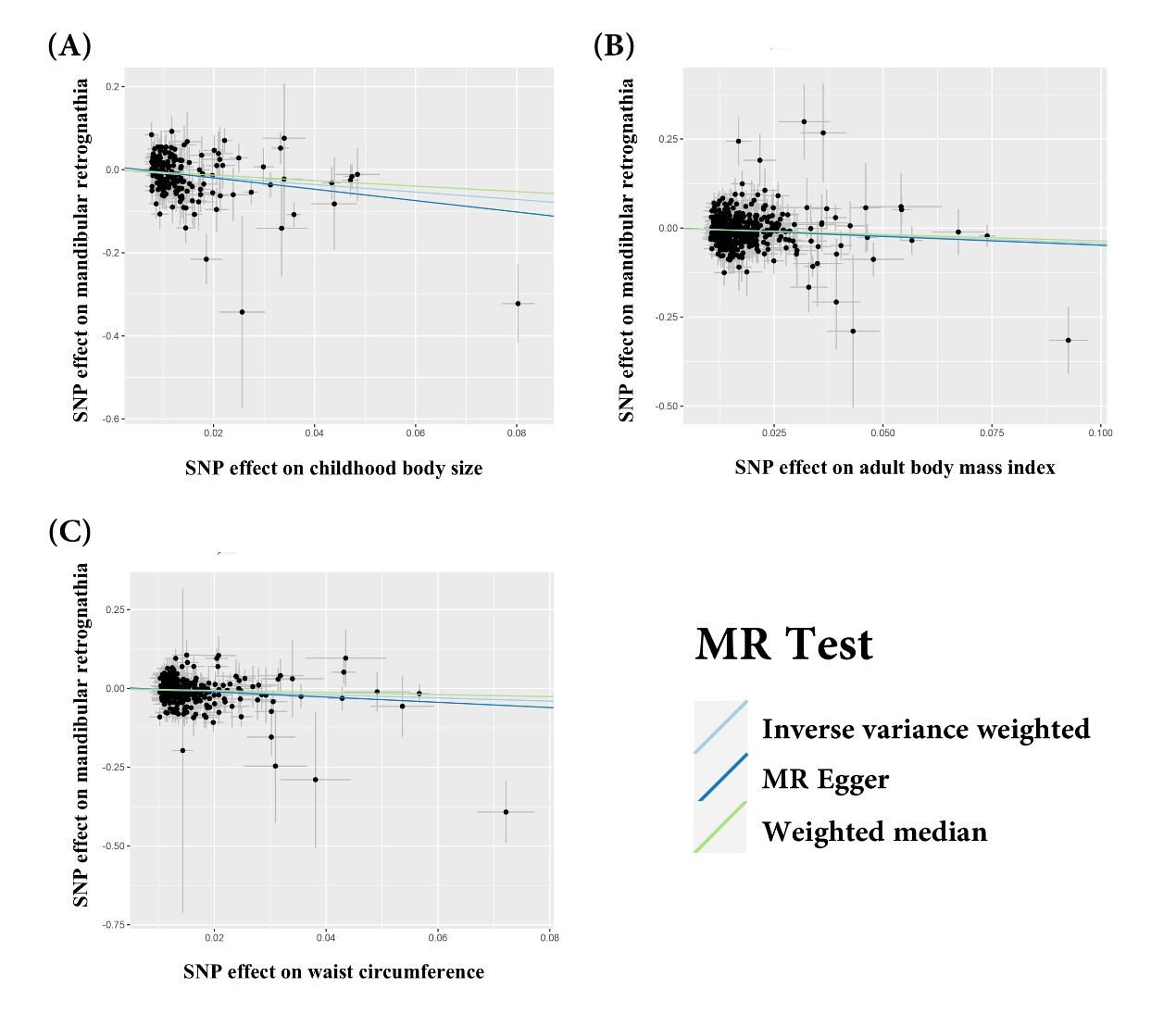


A. Causal estimates for childhood body size on mandibular retrognathia; B. Causal estimates for adulthood body mass index on mandibular retrognathia; C. Causal estimates for waist circumference on mandibular retrognathia.

Supplementary Fig. 2 Funnel plots of estimates from genetically predicted life-course adiposity on mandibular retrognathia.


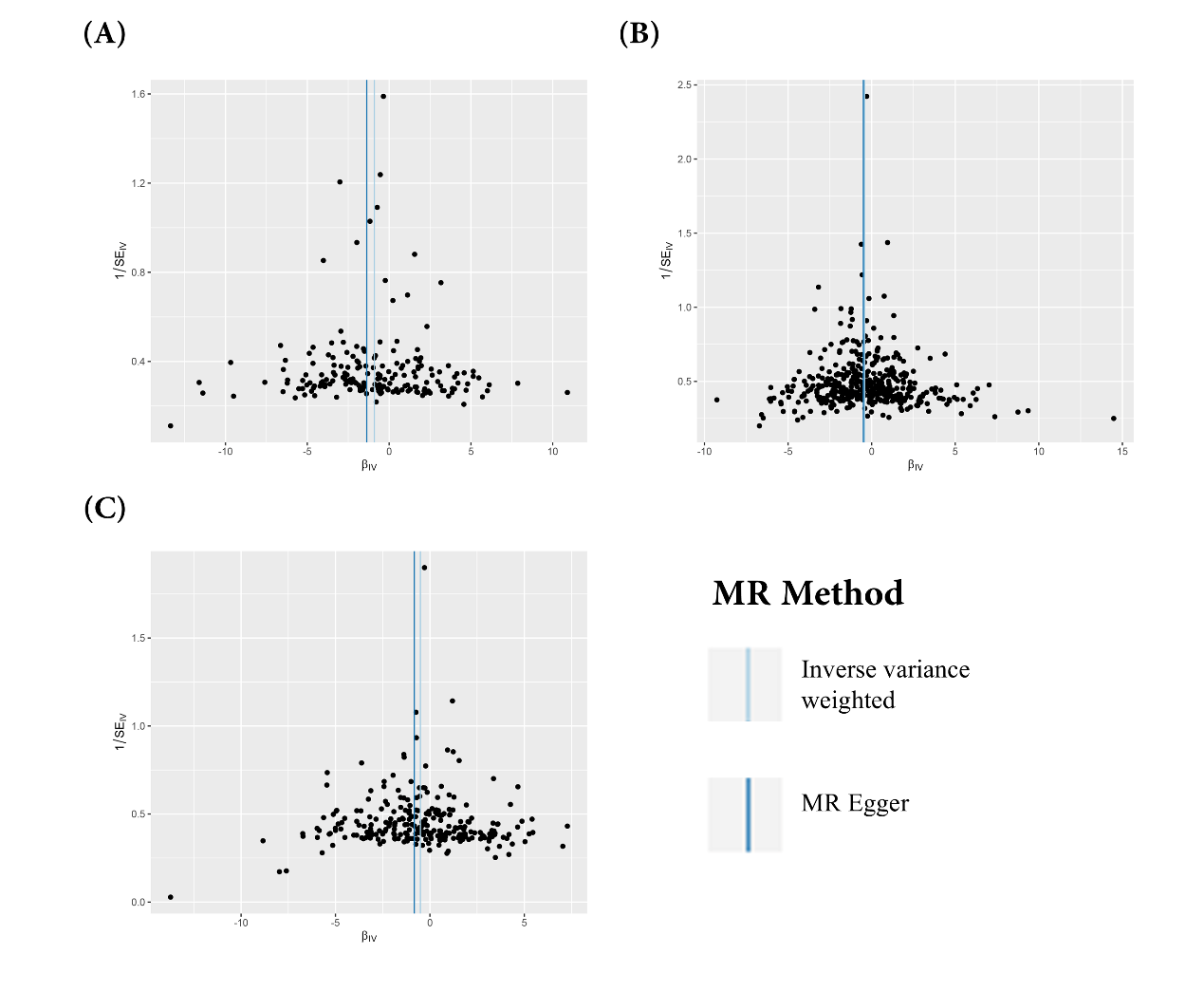


Funnel plots of estimates from (A) genetically predicted childhood body size, (B) adult body mass index, (C) waist circumference.

Supplementary Fig. 3 Leave-one-out plots of estimates from genetically predicted life-course adiposity on mandibular retrognathia using different MR methods.


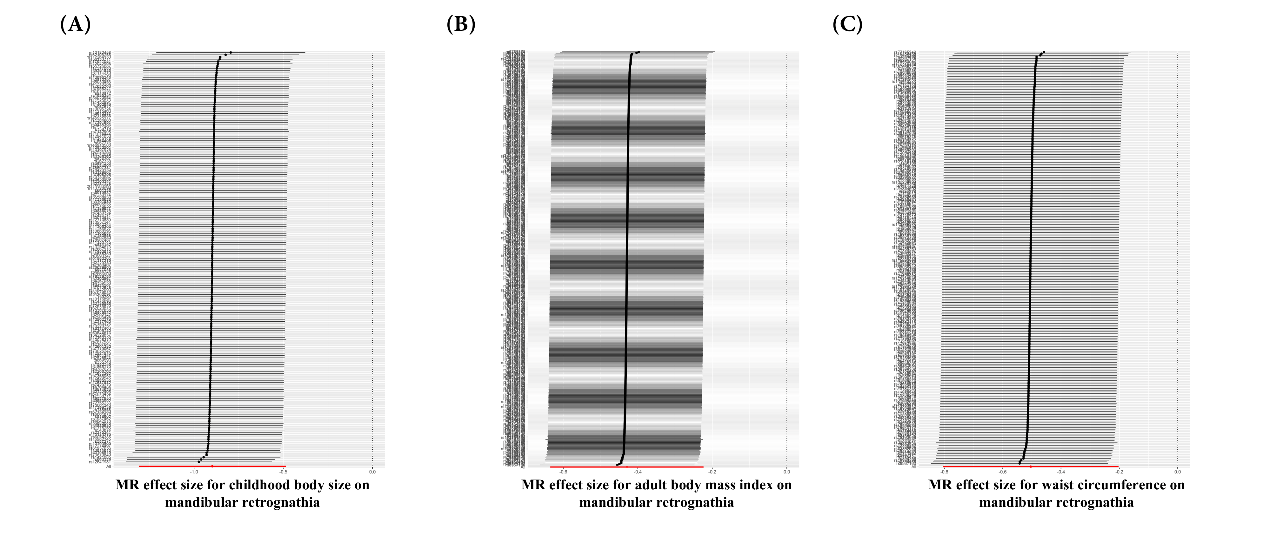


Leave-one-out plots of estimates from(A) genetically predicted childhood body size, (B) adult body mass index, (C) waist circumference.
